# Optimal Quantity of Nano-Silicon for Electrospun Silicon/Carbon Fibers as High Capacity Anodes

**DOI:** 10.3389/fchem.2019.00867

**Published:** 2020-01-17

**Authors:** Renheng Wang, Yiling Sun, Keyu Xiong, Junchao Zheng, Zhengfang Qian, Zhenjiang He

**Affiliations:** ^1^College of Physics and Optoelectronic Engineering, Shenzhen University, Shenzhen, China; ^2^School of Metallurgy and Environment, Central South University, Changsha, China; ^3^College of Environmental Science and Engineering, Donghua University, Shanghai, China

**Keywords:** composite anode, high capacity, silicon, electrospun, nanofiber

## Abstract

In this study, silicon/carbon composite nanofibers (Si@CNFs) were prepared as electrode materials for lithium-ion batteries via a simple electrospinning method and then subjected to heat treatment. The morphology and structure of these materials were characterized by X-ray diffraction (XRD), scanning electron microscopy (SEM), and transmission electron microscopy (TEM). The results show that the structure provides good electrical conductivity and affords sufficient space to accommodate volume expansion during charging/discharging. Furtherly, electrochemical performance tests show that the optimized Si@CNFs have an initial reversible capacity of 1,820 mAh g^−1^ at a current density of 400 mA g^−1^ and capacity retention of 80.7% after 100 cycles at a current density of 800 mA g^−1^. Interestingly, the optimized Si@CNFs have a superior capacity of 1,000 mAh g^−1^ (400 mA g^−1^) than others, which is attributed to the carbon substrate nanofiber being able to accommodate the volume expansion of Si. The SEI resistance generated by the Si@CNFs samples is smaller than that of the Si nanoparticles, which confirms that SEI film generated from the Si@CNFs is much thinner than that from the Si nanoparticles. In addition, the connected carbon substrate nanofiber can form a fiber network to enhance the electronic conductivity.

## Introduction

Lithium-ion batteries (LIBs) are recognized as the green energy of the new century due to their high specific energy, high operating voltage, long cycle life, environmental friendliness, and lack of a memory effect. They have been widely used in the energy storage systems of smartphones, portable audio-visual equipment, small aircraft, electric bicycles, and various small power tools. However, conventional graphite anodes and lithium metal oxide cathodes show relatively low specific energies (~400 Wh kg^−1^ theoretically and ~200 Wh kg^−1^ practically), which cannot meet the growing demand for large-capacity electrochemical energy storage.

The development of new materials with excellent performance and low cost is of great significance for improving battery performance and reducing battery cost. Therefore, research on LIBs should mainly focus on the three aspects of battery cost, battery capacity, and new electrode materials (Lv et al., 2018a,b, 2019; An et al., 2019; Li R. et al., 2019; Li Y. et al., 2019; Wu et al., 2019; Yuan et al., 2019). With the development of anode materials for LIBs, the defects and advantages of various new materials are highlighted. Among all anode materials, silicon (Si) is particularly promising due to its extremely high theoretical capacity (~4,200 mAh g^−1^), low discharge voltage, and natural abundance (Wen et al., 2013; Pan et al., 2015; Tang et al., 2015; Xie et al., 2015; Casimir et al., 2016; Wang and Yang, 2017; Yamaguchi et al., 2017; Zuo et al., 2017; Fang et al., 2019). However, Si as an anode material undergoes significant volume expansion during the Li^+^ ion lithiation/delithiation process, leading to particle fracture and loss of capacity (Datta et al., 2011; Zhong et al., 2014; Huang et al., 2016; Jeong et al., 2016; Lin et al., 2016; Yang et al., 2016; Kim et al., 2017).

One generally accepted strategy to overcome this volume expansion is to synthesize nanoscale Si particles. Kim devised a simple, reproducible and cost-effective method for fabricating nanoporous Si flakes that demonstrate high electrochemical performance (Kim et al., 2017). Chen et al. reported an effective method for synthesizing Si nanotubes that exhibit significantly improved electrochemical properties (Wen et al., 2013). However, the above method of nanocrystallization only partially alleviates this problem and meanwhile improves the cycle performance to only a limited extent. Nowadays, silicon/carbon (Si/C) is being widely synthesized for use as an anode in LIBs because it combines the advantages of both carbon and Si and has been proposed to a very promising material to replace the commercially available graphite. Nathalie et al. have developed a two-stage LCVP to synthesized core-shell Si/C nanoparticles (Sourice et al., 2016). Sun reported a 3D Si/CNCS nanocomposite consisting of a CNC three-dimensional interconnected conductive network and a unique Si/CNC hollow sphere structure. The Si/CNCS nanocomposite can effectively alleviate volume expansion and improve electronic conductivity (Yue et al., 2016). Additionally, Si/C composite nanofibers (Si@CNFs) can be prepared for use as anodes in LIBs through a simple and low-cost method combining electrospinning and subsequent thermal treatment (Ji and Zhang, 2009; Hwang et al., 2012; Wu et al., 2014, 2016).

In this work, Si@CNFs materials were fabricated via a facile electrospinning technique. The Si@CNFs display an initial reversible capacity of 1,820 mAh g^−1^ at a current density of 400 mA g^−1^ and capacity retention of 80.7% after 100 cycles at a current density of 800 mA g^−1^ and exhibit a superior capacity of 1,000 mAh g^−1^ at a current density of 400 mA g^−1^. The SEI resistance generated by the Si@CNFs samples is smaller than that of the Si nanoparticles, which confirms that SEI film generated from the Si@CNFs is much thinner than that from the Si nanoparticles. In addition, the connected carbon substrate nanofiber can form a fiber network that enhances the electronic conductivity. The crystal structure was measured by X-ray diffractometer (PAnalytical X'Pert ProMRD, Holland). The surface morphology was analyzed by field emission scanning electron microscopy (SEM, JEOL, JSM-5612LV). The microstructure was observed by transmission electron microscopy (TEM, JEOL-JEM-2100F). The cycling performances of Si nanoparticles and Si@CNFs are evaluated. The charge transfer resistances of Si nanoparticles and Si@CNFs anodes are analyzed by electrochemical impedance spectroscopy (EIS).

## Experimental Methods

**Figure 1:**
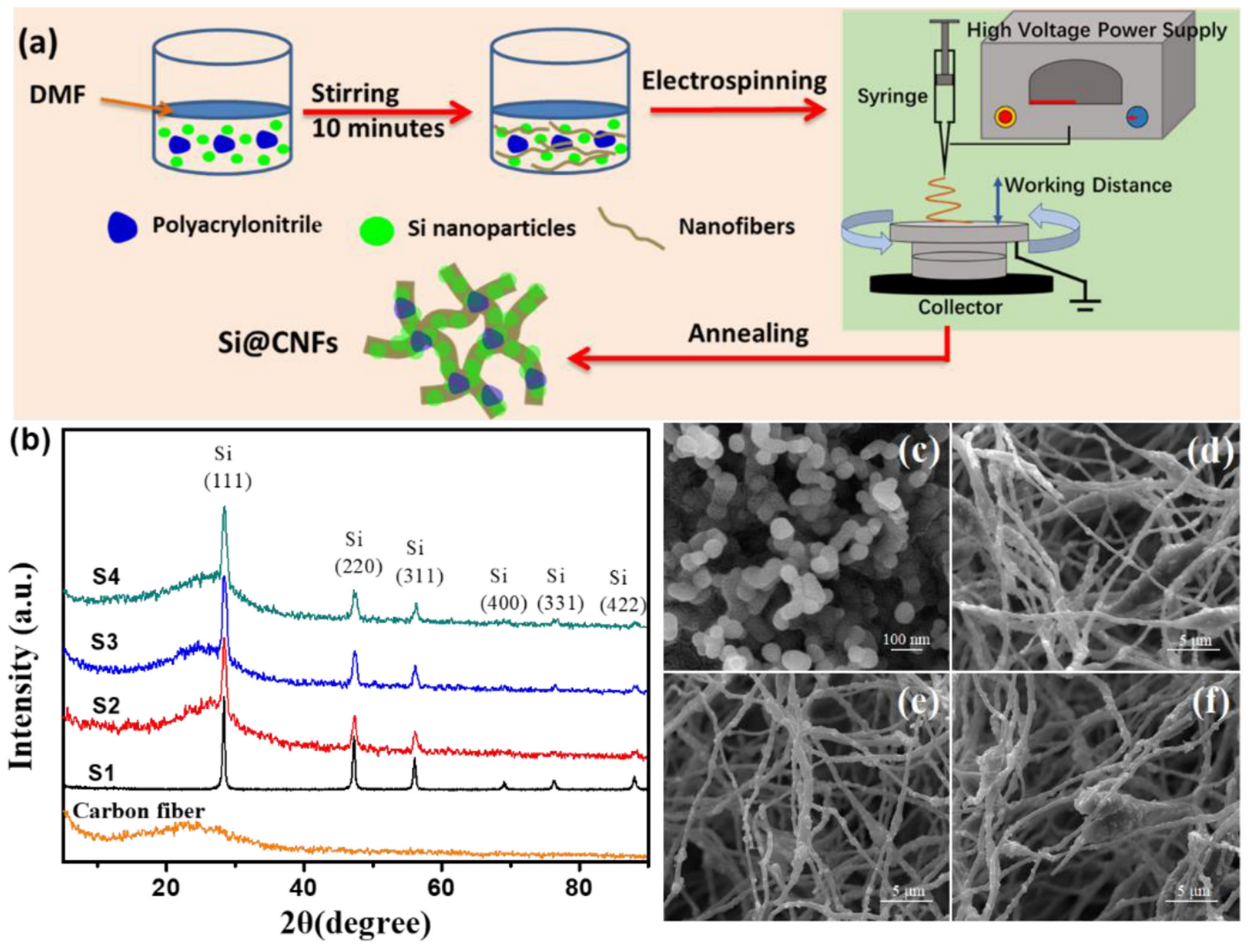
**(a)** Schematic illustration of Si@CNF preparation. **(b)** XRD patterns of carbon nanofibers, Si nanoparticles (S1), and Si@CNFs (S2, S3, and S4); SEM images of **(c)** S1, **(d)** S2, **(e)** S3, and **(f)** S4 samples.

The preparation of Si@CNFs was carried out through the facile electrospinning method shown in Figure 1a. Specifically, first, 1 g polyacrylonitrile (0.15 M), purchased from Sigma Aldrich, was dissolved in 10 mL N, N-Dimethylformamide (DMF). Meanwhile, 0.10, 0.20, or 0.30 g of Si nanoparticles was mixed well with the above solution, followed by stirring and keeping the mixed solution under sonication for 10 min. This solution was added to the nanofibers for electrospinning using an electrospinning device. The operating parameters were as follows: an applied voltage of direct current (DC) 25 kV (Dongwen, P503-1ACDF), working distance of 15 cm between the collector (aluminum foil) and solution syringe needle, and pumping rate of 2 mL h^−1^. The prepared composite nanofibers were annealed in air at 260°C for 2 h and in an argon atmosphere at 600°C for 6 h to obtain Si@CNFs. The Si nanoparticles were labeled as S1. The Si@CNFs prepared using 0.10, 0.20, and 0.30 g Si nanoparticles correspond to the names S2, S3, and S4 in this article, respectively.

The electrochemical performances of all samples were tested using 2025-type coin half-cells. Celgard 2400 microporous polypropylene membrane was used as the separator, and the lithium metal sheet was used as the negative electrode. One mol L^−1^ LiPF_6_/EC+EMC+DMC (by weight ratio, 1:1:1) was used as the electrolyte. Si nanoparticles, polyvinylidene fluoride (PVDF), and acetylene black were weighed according to the mass ratio of 8:1:1 and thoroughly mixed and ground, an appropriate amount of N-methylpyrrolidinone (NMP) was added to prepare an electrode slurry, and the slurry was uniformly coated on the copper foil with an applicator. After drying in a vacuum oven at 120°C for 15 h, it was punched into a pellet with a mass of 2.0 mg. The Si@CNFs were used directly as an electrode film. The CR2025 button cell was assembled in a glove box filled with argon at a relative humidity of less than 5% and an oxygen pressure of less than 1.0 ppm. After 12 h of activation, the electrochemical performance tests were performed in a voltage range of 0.01–2.0 V from 400 to 4,000 mA g^−1^.

## Results and Discussion

Figure 1b shows the typical XRD patterns of all samples. The pure carbon fiber shows a diffraction peak at 2θ = 25.0°, belonging to the (002) plane (Li et al., 2014). However, the peak of the nanofiber is weak and broad, resulting from disordered carbon. The diffraction peaks of Si@CNFs are at 2θ values of 28.5°, 47.4°, 56.3°, 69.2°, 76.8°, and 88.3°, respectively, corresponding to the (111), (220), (311), (400), (331), and (422) peaks of Si crystals in the nanofibers (Chen et al., 2011; Xie et al., 2014). It is obvious that the fluctuations in the XRD patterns of S2, S3, and S4 at 2θ of 5°-40° are caused by the action of carbon nanofibers. In addition, under these synthesis conditions, the synthesis procedures have no impact on the crystal structure of silicon.

**Figure 2:**
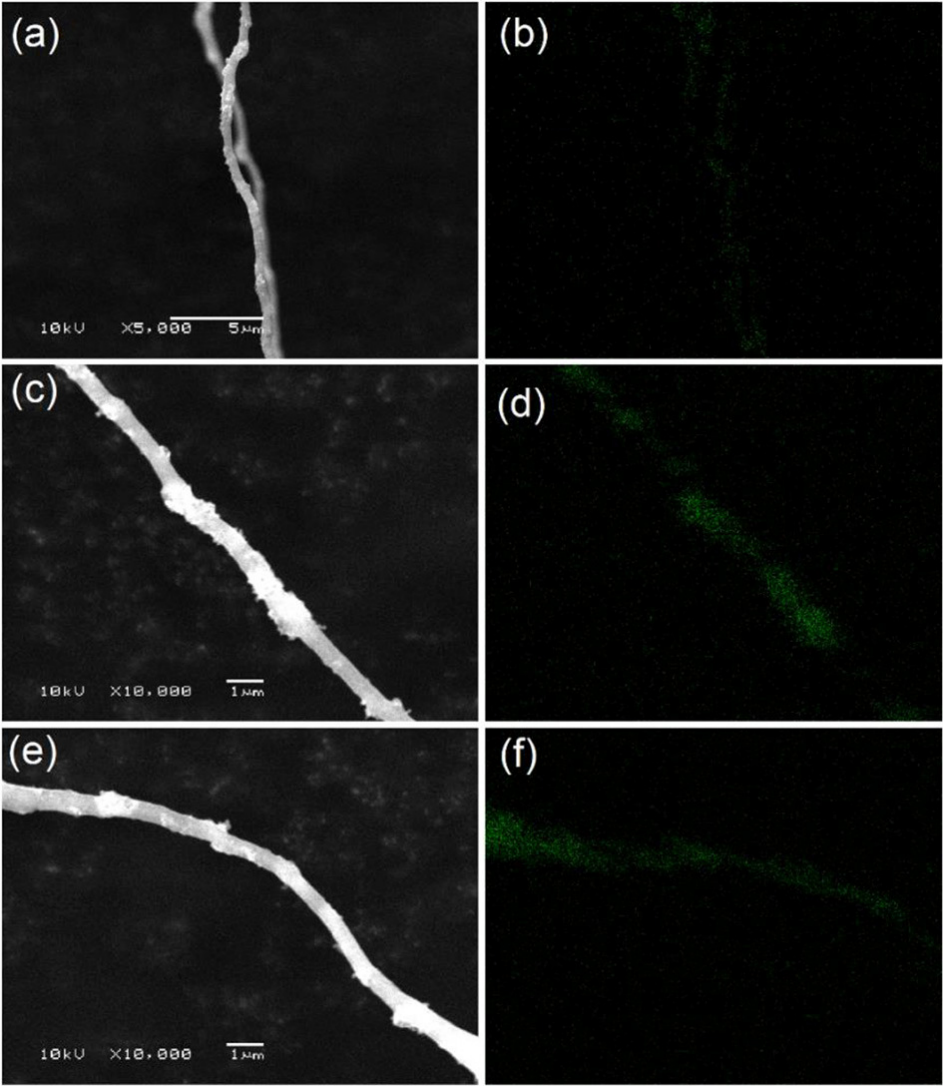
EDS images of sample S2 **(a,b)**, sample S3 **(c,d)**, and sample S4 **(e,f)**.

Figures 1c–f illustrates SEM images of Si nanoparticles and electrospun Si@CNFs. The size of Si nanoparticles is about 30–60 nm, as shown in Figure 1c, as they easily form agglomerated particles. However, the independent fiber skeleton of Si@CNFs has diameters of 300–500 nm, as displayed in Figures 1d–f. The net structure of the interconnected carbon network can be beneficial in improving the electrode surface and providing fast ion-conducting channels. The structure is expected to provide good electrical conductivity and afford sufficient space to accommodate volume expansion during charging/discharging. Besides, a compact fiber can avoid the formation of an unstable solid electrolyte interphase (SEI) layer on the surface of Si nanoparticles (Wu et al., 2016). The distributions and elemental mappings of Si in Si@CNFs were measured by energy dispersive spectrometer (EDS), as shown in Figure 2. The Si component is uniformly distributed in the three matrixes along both the radial and axial directions.

**Figure 3:**
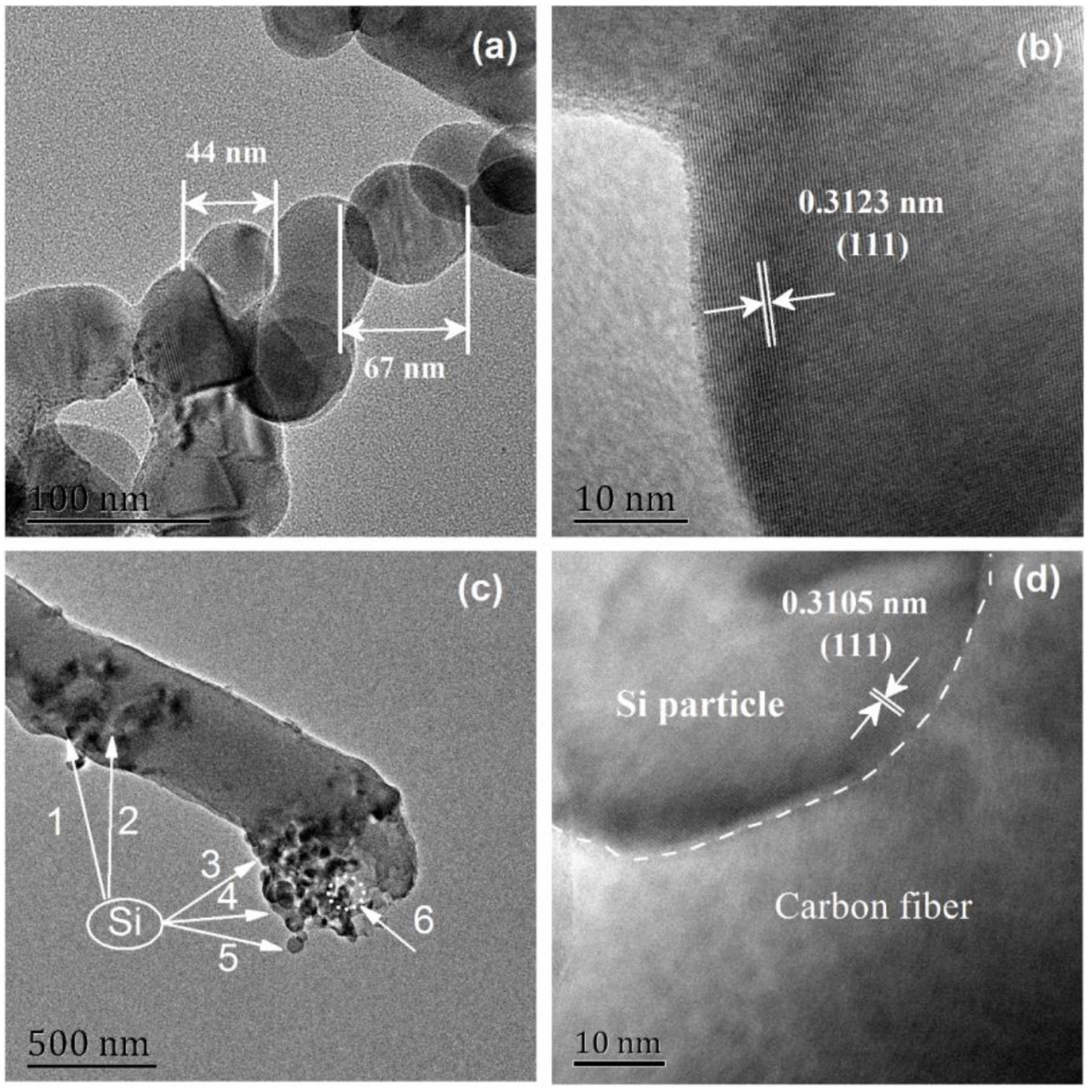
HRTEM images of Si nanoparticles (S1) **(a,b)**, Si@CNFs (S3) **(c,d)**.

Figure 3 displays the microstructures of Si nanoparticles (S1) and Si@CNFs (S3). The clear crystal grain edge and the complete crystallinity of the 0.3123 nm lattice spacing are clearly seen in Figures 3a,b. The lattice spacing is derived from Si (111), with different lattice stripes extending to the particle boundaries. As seen in Figures 3c,d, the structure of the carbon nanofibers looks like a fishnet. The Si in S3 is well wrapped up by carbon nanofibers, providing enough space to accommodate huge volume changes during charging and discharging (Zhou et al., 2016; Wang et al., 2017a,b). It can be concluded that electrospinning is an effective technique for synthesizing S3.

**Figure 4:**
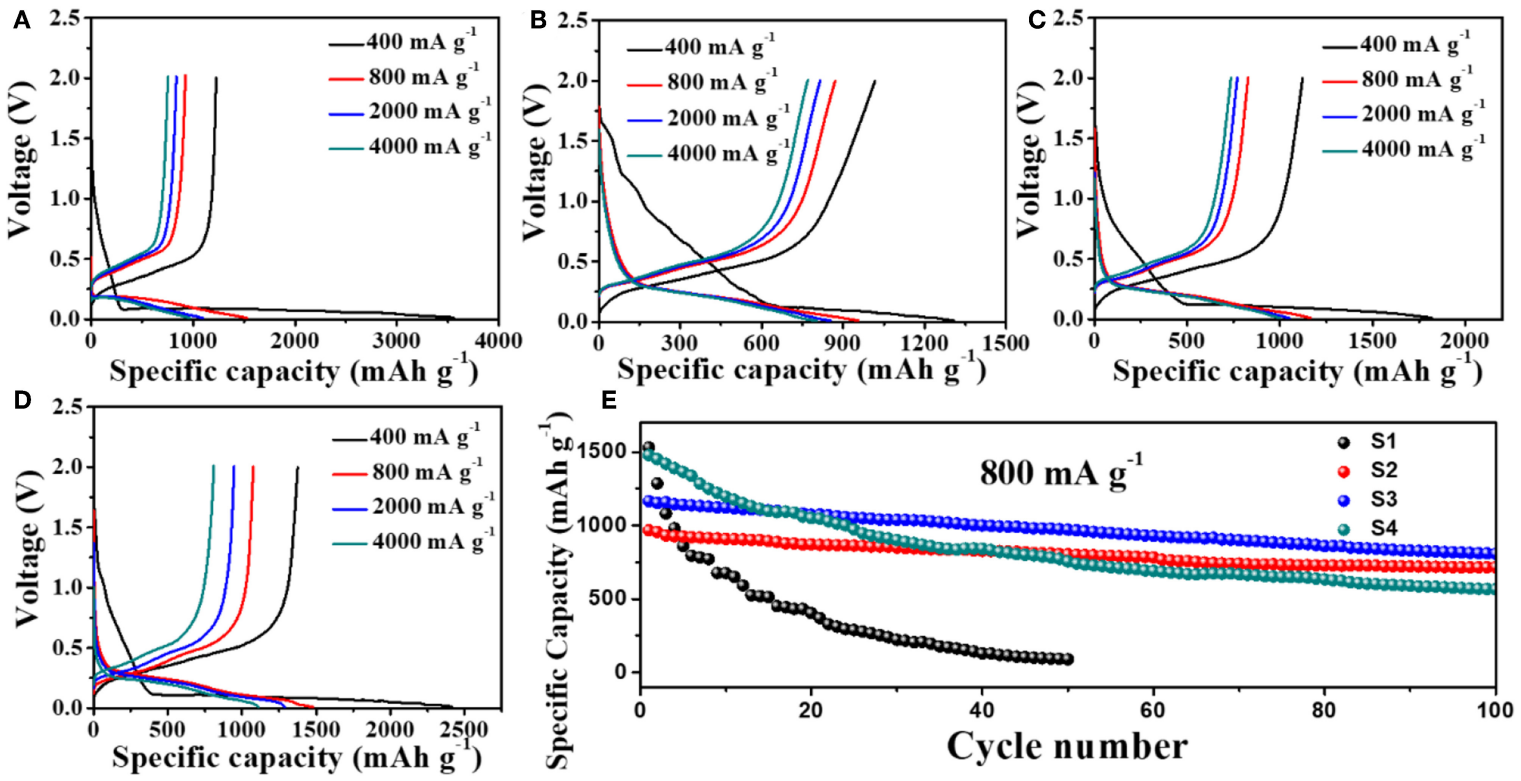
Initial charge/discharge curves of **(A)** Si nanoparticles (S1) and Si@CNFs **(B)** S2, **(C)** S3, **(D)** S4, and **(E)** cycling performances of Si nanoparticles (S1) and Si@CNFs (S2, S3, S4) between 0.01 and 2.0 V at 25°C.

Figure 4 and Table 1 show the electrochemical performances of Si nanoparticle and Si@CNF half-cells at different current densities. Figures 4A–D display the initial charge and discharge of four samples. It can be seen that the initial charge-discharge capacities of Si@CNF samples are higher than that of the Si nanoparticle sample at all current densities. When the current density is 400 mA g^−1^, the capacity of the Si nanoparticles is 3,555 mAh g^−1^, but the capacity declines sharply to 966 mAh g^−1^ when the current density increases to 4,000 mA g^−1^, only 27.2% of the initial capacity. However, Si@CNFs (take sample S3 as an example) have better rate performances. The initial capacities are 1,820, 1,166, 1,050, and 1,000 mAh g^−1^ at 400, 800, 2,000, and 4,000 mA g^−1^, respectively. It is clear from Figure 4E that Si nanoparticles still delivered the worst cycle performance at 800 mA g^−1^, only 5.8% of the initial capacity after 50 cycled charging/discharging processes. However, Si@CNFs remarkably improved the cycle stability vs. Si nanoparticles. After 100 cycles, the discharge capacities of sample S2, S3, and S4 represent 89.3, 80.7, and 50.8% of the initial capacity, respectively. Obviously, a reduction in the capacity of Si@CNFs will occur with a decrease in the silicon content, which is due to the higher specific capacity of silicon nanoparticles than of carbon nanofibers. However, the cycle stability of Si@CNFs dropped with an increase in silicon content. Therefore, ensuring that there is an appropriate amount of silicon nanoparticles wrapped in carbon nanofibers is a key point for improving the rate capacity and cycling performance at the same time. Here, sample S3 displays the best comprehensive performance.

**Table 1:** Rate performances of silicon nanoparticles and Si@CNFs.

**Sample**	**Discharge capacity at 400 mA g^**−1**^/** **(mAh g^**−1**^)**	**Discharge capacity at 800 mA g^**−1**^/** **(mAh g^**−1**^)**	**Discharge capacity at 2,000 mA g^**−1**^/** **(mAh g^**−1**^)**	**Discharge capacity at 4,000 mA g^**−1**^/** **(mAh g^**−1**^)**
S1	3,555	1,531	1,096	966
S2	1,306	956	852	802
S3	1,820	1,166	1,050	1,000
S4	2,413	1,479	1,289	1,112

**Figure 5:**
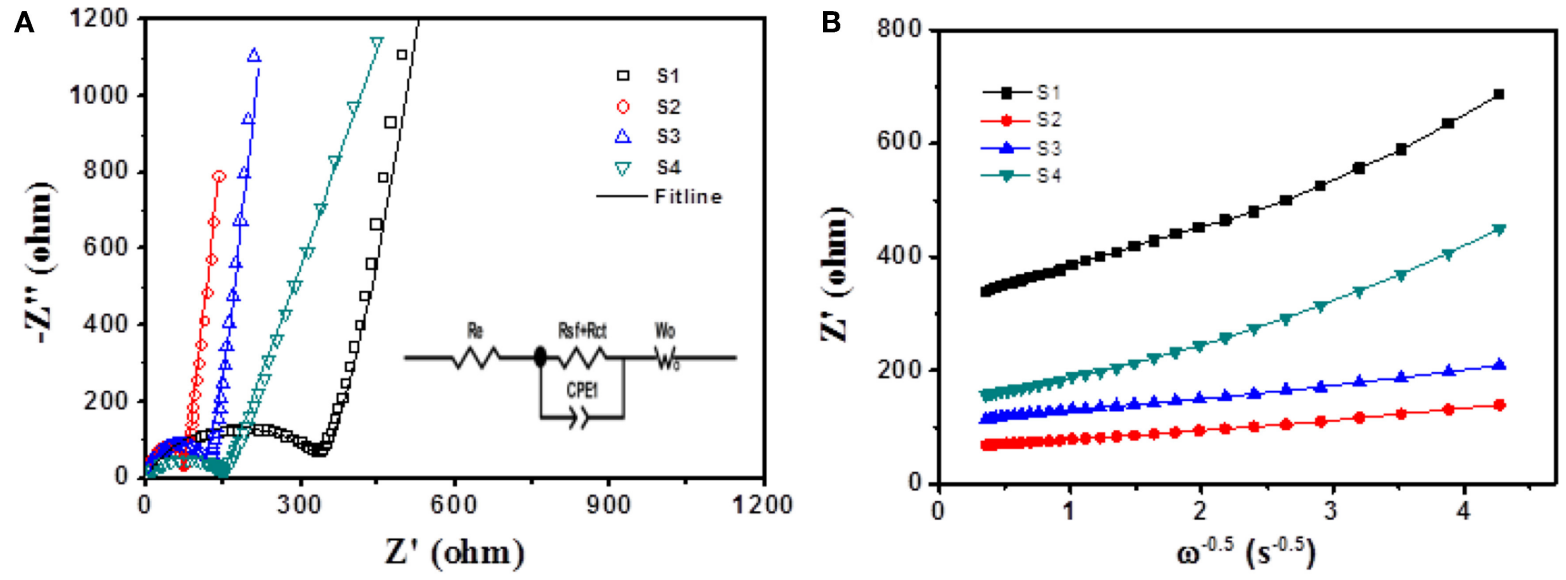
**(A)** Electrochemical impedance spectra of Si nanoparticles (S1) and Si@CNFs (S2, S3, S4); the inset illustration is the equivalent circuit diagram as fitted by ZView software. **(B)** Plots of Z′ vs. ω^−0.5^ for Si nanoparticles and Si@CNFs.

In order to achieve an in-depth understanding of the superior electrochemical performance of Si@CNFs, EIS measurements were used to evaluate the electrode kinetics factors of Si nanoparticles and Si@CNFs, as shown in Figure 5. The inset illustration is the equivalent circuit diagram fitted by ZView software (Wang et al., 2017a,b). From Figure 5A, it can be found that the EIS plots were composed of three parts: a semicircular arc at high frequency corresponding to SEI resistance (R_sf_), a semicircle at intermediate frequency related to charge-transfer resistance (*R*_ct_), and a straight line at low frequency connected to the Warburg diffusion process of lithium ions into the electrode material (*W*_O_). *R*_e_ represents solution resistance, and *CPE* is attributed to the double-layer capacitance and the passivation film capacitance. The corresponding resistance values are displayed in Table 2. The *R*_e_ values of all samples are very similar and small. The *R*_sf_ and *R*_ct_ values of the Si@CNFs samples are smaller than those of the Si nanoparticles, which confirms that the SEI film generated from the Si@CNFs was much thinner than that from the Si nanoparticles. This may prove that the Si@CNFs can effectively reduce the diffusion resistance and charge transfer resistance, thus making it easier for Li^+^ to migrate/embed in the lattice. It is noted that *R*_ct_ increases from 68.0 (S2) to 332.8 Ω (S1) when the Si content is increased. The decrease in *R*_ct_ may result from the enhanced electron and ionic conductivity of carbon nanofibers. In detail, the existence of carbon nanofibers can reduce *R*_ct_, but the influence becomes weak when the silicon content reaches a certain value. Furthermore, the specific capacity of Si@CNFs decreased sharply when the silicon content was reduced. In addition, the exchange current densities (*i*^0^) were calculated by the formula (*i*^0^ = *RT*/*nFR*_ct_), and the results are shown in Table 2. The S2 sample has the highest exchange current density (3.78 × 10^−4^ mA cm^−2^) in all samples, and S3 also has a higher exchange current density (2.49 × 10^−4^ mA cm^−2^) than S1 (7.72 × 10^−5^ mA cm^−2^) and S4 (1.78 × 10^−4^ mA cm^−2^). Thus, the EIS results indicate that the Si@CNFs possesses good charge transfer kinetics within the electrode.

**Table 2:** Impedance parameters of Si nanoparticles and Si@CNFs.

**Sample**	**R_**Sf**_ (Ω)**	**R_**ct**_ (Ω)**	***i^**0**^* (mA cm^**−2**^)**
S1	4.22	332.8	7.72 × 10^−5^
S2	3.51	68.0	3.78 × 10^−4^
S3	3.29	103.1	2.49 × 10^−4^
S4	3.86	144.5	1.78 × 10^−4^

Figure 5B shows the relationship between the real axis and the square root of the lower angular frequency (Xie et al., 2014; Wang et al., 2017b). The slopes of the straight lines indicate the values of the Warburg impedance coefficient (σ_w_). It is noted that the lithium-ion diffusion process resistance of sample S2 is lower than those of the other samples, indicating that its net structure has the shortest diffusion length.

## Conclusion

Si@CNFs materials have been synthesized by electrospinning technology, and the crystal structures of Si@CNFs are similar to those of Si nanoparticles. The Si nanoparticles in Si@CNFs are well wrapped up by carbon nanofibers. This network structure may provide good electrical conductivity, afford sufficient space to accommodate volume expansion during charging/discharging, enhance Li^+^ diffusion, and reduce charge-discharge resistance. It is interesting that the optimized Si@CNFs hold a superior capacity of 1,000 mAh g^−1^ (400 mA g^−1^), which is attributed to the carbon substrate nanofiber being able to accommodate the volume expansion of Si. The SEI resistance generated by Si@CNFs is lower than that of others. However, too much carbon content will decrease the specific capacity of Si@CNFs. Sample S3 displays the best comprehensive performance and delivers a reversible capacity of 1,820 mAh g^−1^ between 0.01 and 2.0 V at 400 mA g^−1^, corresponding to 80.7% of the 100-cycle initial discharge capacity at 800 mA g^−1^. We believe that Si@CNFs are a promising anode material for LIBs.

## Data Availability Statement

All datasets generated for this study are included in the article/supplementary material.

## Author Contributions

RW and ZH designed and engineered the samples and performed the experiments. RW, ZH, and ZQ performed the data analysis. ZH, YS, ZQ, and RW wrote the paper. All authors contributed to the theoretical analysis and the general discussion.

### Conflict of Interest

The authors declare that the research was conducted in the absence of any commercial or financial relationships that could be construed as a potential conflict of interest.
